# How reliable is anamnestic data in predicting the clinical relevance of house dust mite sensitization?

**DOI:** 10.1007/s00405-021-06862-x

**Published:** 2021-05-21

**Authors:** Anna S. Englhard, Martin Holzer, Katharina Eder, Donata Gellrich, Moritz Gröger

**Affiliations:** Department of Otorhinolaryngology-Head and Neck Surgery, Klinikum der Universität München, Marchioninistr. 15, 81377 Munich, Germany

**Keywords:** House dust mite, Sensitization, Allergy, Provocation test

## Abstract

**Purpose:**

For perennial inhalant allergens such as house dust mite (HDM), the German guideline on allergen-specific immunotherapy explicitly recommends provocation testing. This procedure is time-consuming, expensive, and potentially dangerous for the patient. Recently it has been discussed whether provocation tests are really necessary for diagnosing allergy to *Dermatophagoides* species. Thus, our study aimed to analyze the concordance between nasal provocation tests with *Dermatophagoides* species and anamnestic data.

**Methods:**

We retrospectively analyzed the concordance between patients’ histories including self-reported symptom scores and the results of provocation testing in 471 individuals with proven sensitization to *Dermatophagoides* species.

**Results:**

248 patients had a positive nasal provocation test (NPT) result to *Dermatophagoides* species and 223 individuals a negative NPT result. Patients allergic to HSM suffered significantly more often from atopic dermatitis (14% vs. 7%, *p* = 0.046) and more from asthma (20% vs. 19%, *p* = 0.851). Moreover, individuals with clinically silent sensitization complained less about nasal secretion (37% vs. 45%, *p* = 0.244) but significantly more about nasal dryness (46% vs. 34%, *p* = 0.046) whereas rates of nasal airway obstruction, ocular complaints and sleep quality were comparable in both groups. Allergic patients reported more often perennial (34% vs. 30%, *p* = 0.374) and location-dependent (39% vs. 31%, *p* = 0.090) symptoms. However, the discrepant prevalence of atopic dermatitis was the only statistically significant difference between both groups.

**Conclusion:**

Despite slight differences between both patient groups, clinical data are not sufficient to distinguish between silent sensitization and clinically relevant allergic rhinitis to HDM. Therefore, nasal provocation testing remains the gold standard for assessing clinical relevance in patients sensitized to *Dermatophagoides* species.

## Introduction

House dust mite (HDM) is the most important indoor allergen and one of the three most relevant aeroallergens responsible for the development of allergic symptoms such as allergic rhinitis or allergic asthma worldwide [1, 2]. Data about the prevalence of allergy to HDM are inconsistent. The GA^2^LEN study elicited relevant regional discrepancies in the prevalence of sensitization to HDM in adults throughout Europe reaching rates from 9.3% in Sweden to 30.6% in Spain [3]. From a clinician’s point of view, the incidence of allergic rhinitis (AR) upon exposure to HDM is obviously more relevant than clinically silent sensitization. This issue was addressed by the GA^2^LEN skin test study II, published by Burbach et al. in which the authors differentiated between silent sensitization and clinically relevant sensitization assessed by experienced allergologists with the skin prick test (SPT), the history and further tests such as provocation testing in different European countries with different inhalant allergens. In case of HDM, they found that at least 80% of the participants with positive SPT reaction had a clinical relevant sensitization to HDM in most investigated countries. Only in France, Finland and Austria they revealed clinically relevant sensitization rates of less than 65% [4]. These findings were corroborated by a study of Blomme et al. in which the prevalence of silent sensitization and allergy to HDM in an unselected population in Belgium was analyzed by SPT and an interview of the participants about possible allergic symptoms. They found a prevalence of silent sensitization to HDM in 25.9% and symptoms of AR caused by HDM in 17.1% of the participants [5].

It has been demonstrated that untreated HDM allergy of the upper airway has the risk of transforming into a disease of the lower airways with full symptoms of allergic asthma [2, 6–8]. These results clearly underline the socio-economic impact of HDM allergy as a relevant disease generating high costs for the health care systems and severe complaints for lots of patients.

When diagnosing patients with allergic symptoms, a detailed medical history and thorough physical examination are the important first steps [9, 10]. This procedure should be followed by SPT [10]. Patients suffering from one single seasonal allergy, for example to grass pollen, can be diagnosed by history and positive SPT alone in case they report about reproducible allergic symptoms occurring every year at the same specific time [11]. In contrast, perennial allergens like HDM frequently cause unspecific symptoms throughout the year in divergent intensity [12]. For this reason it can be difficult to match the patients’ complaints and the exposition to the allergen only based on the history. As a consequence, further diagnostic steps such as determination of specific immunoglobulin E (sIgE) to Der p 1, Der p 2 alone or in combination with SPT might be necessary [9, 11]. However, both, SPT and sIgE may only detect sensitization, which is not equivalent to a clinically relevant allergy as underlined above. Thus, the German guideline on allergen-specific immunotherapy [13] in IgE-mediated allergic diseases explicitly recommends provocation testing for perennial inhalant allergens such as HDM before initiating allergen immunotherapy [13, 14]. In case of AR, this approach usually takes the form of specific nasal provocation testing (NPT) [15]. As NPT is a relatively complex procedure and most clinicians even do not have the technical option to perform it, an easier way to differentiate between clinically relevant and irrelevant HDM sensitizations is desirable. Although there are several studies analyzing the correlation between NPT and sIgE-serology in HDM sensitization [16, 17], there is to our knowledge no data in literature on the concordance between anamnestic data and NPT in HDM sensitized patients. Therefore, there is a need of an evidence-based study to confirm the general assumption that medical history is too inconsistent in HDM patients making allergen challenge testing necessary.

Thus, the purpose of our study was to compare the medical history of patients with clinically silent sensitization and those with clinically relevant allergy to HDM. We aimed to identify specific questions, which could facilitate a differentiation of both patient groups based on medical history. This could help to avoid time-consuming and risky procedures like NPT at least in some patients.

## Materials and methods

### Study population

Patients presenting at our institution, the Department of Otorhinolaryngology of the Ludwig-Maximilians-University, Munich, Germany, received the SX1-screening test [18], if an allergic cause of their complaints was reasonable. The SX1 is an in vitro screening test for inhalant allergy (Phadiatop, Pharmacia Uppsala, Sweden). The test is based on the Fluoroenzyme immunoassay-Test (CAP-FEIA). Specific IgE antibodies to different inhalant allergens like, HSM, birch, grass, rye, cat, dog, mugwort and cladosporium are detected simultaneously. Our group was able to demonstrate the value of this test for screening some time ago [19]. In case of a positive screening test result or in case of a negative test result but persisting clinical suspicion (location-dependent symptoms, symptoms in beds, allergic asthma), the patient was recommended to attend the allergologic consultation hours for further clarification. Within this appointment, each patient had to fulfill a detailed questionnaire, comprising standardized questions of the most relevant symptoms over the last 7 days adopted from the Rhinoconjunctivitis Quality of Life Questionnaire (RQLQ) [20, 21] in German language. The adapted questionnaire we used for the presented study contains of two parts: the first part consists of a general allergologic history, e.g. questions about location dependency, allergic family members, seasonal symptoms, home environment including indoor pants, carpets, age of mattress, use of encasings, history of allergic shock, history of asthma, and history of food intolerance. The second part comprises standardized questions of the most relevant symptoms over the last 7 days. Patients were asked to rate their symptoms on a scale from 0 to 3 (0 = no complaints, 1 = slight complaints, 2 = mild complaints, 3 = severe complaints). The questionnaire includes nasal symptoms, reduced physical performance, sore throat, headache, sleep dependent symptoms, ocular complaints, asthmatic symptoms, complaints related to food intolerance, dermatologic symptoms, and emotional complaints.

In addition to this questionnaire, the attending physician took the history followed by SPT, blood test and in case of suspected HDM allergy by NPT. The questionnaire and the patient’s history were saved together with the results of the examinations as a complete data set for each patient. Consequently, this procedure generated a preselected but suitable database containing many data sets of mono- or polysensitized but also of mono- and polyallergic patients. For the purpose of the presented study, this allergy database was scanned retrospectively to identify patients with proven sensitization to HDM based on a positive SPT result and/or a positive sIgE measurement. All subjects included had underwent SPT and allergen-specific provocation as routine in vivo tests and total IgE and allergen-specific IgE measurements in serum as in vitro tests. The study was based on anonymized data and approved by the local ethics committee. All patients provided their written informed consent for the use of their data for scientific purposes.

### Skin prick testing

SPT was performed with a solution for HDM testing (ALK-Abelló, Copenhagen, Denmark) according to published guidelines [22]. We performed SPT to birch, hazel, alder, ash, tomithy grass, rye mugwort, pellitory, ragweed, *Dermatophagoides pteronyssinus*, *Dermatophagoides farina*, dog, cat, Alternaria and Aspergillus.

### Fluorescence enzyme immunoassay

IgE reactivity to natural allergen extract (d1) and allergen components Der p 1 and Der p 2 was measured using fluorescence enzyme immunoassay (UniCAP-FEIA, ThermoFisher Scientific, Freiburg, Germany) with a commercially available test kit (Phadia Diagnostics, Uppsala, Sweden). In case of a positive SPT result to allergens other than HDM, specific IgE antibodies to the corresponding native extracts and allergen components were also measured. Results were reported as concentrations (kU/L). The positive cutoff value was > 0.35 kU/L as suggested by the manufacturer.

### Nasal provocation testing

In accordance with current guidelines, all patients sensitized to HDM underwent NPT [14]. The protocol used in this study has been previously described [23]. Following the German position paper regarding NPT, following carence times were respected: DNCG, Nedocromil 3 days, nasal corticosteroids 7 days, oral corticosteroids 7 days, nasal antihistamines 3 days, oral antihistamines 3 days, nasal alpha-adrenergics 1 day, tricyclic psychotropic drugs 3 days [24, 25]. Following exclusion criteria were applied: acute infectious diseases of the nose or the paranasal sinuses, acute allergic reactions in other parts of the body, severe general illness, nasal operations less than 2 months ago, treatment with beta-blockers, and vaccination 1 week before the NPT. Relative contraindications were pregnancy, age below 5 years and extremely high grade of sensitization. Patients with only partly controlled or uncontrolled asthma (following the GINA guidelines [26] including FEV1 < 80%, symptoms more often than twice a week during the day, nocturnal symptoms, emergency medication necessary more often than twice a week) were transferred to the department of pulmonary medicine prior to NPT. We performed nasal endoscopy in all patients before they underwent NPT. Prior to NPT patients had 30 min time to adapt to the climatic circumstances of the examination room. Patients received two puffs of challenge solution. After each application individuals waited for 10 min before rhinomanometry was performed and their symptoms were evaluated. Patients were diagnosed to have an unspecific challenge reaction in case that after the administration of allergen-free solution a flow reaction of more than 20% was seen. First, active anterior rhinomanometry (RhinoSys, Happersberger otopront GmbH, Hohenstein, Germany) was performed to obtain a baseline measurement. It was repeated after the administration of allergen-free solution (LETI Pharma GmbH, Ismaning, Germany) and finally after the application of solution containing the allergen (*D. pteronyssinus* or *farinae*, 100 HEP/mL; LETI Pharma GmbH, Ismaning, Germany) via a nasal spray pump. As monosensitization to *D. farinae* is rare; in general, we performed NPT with *D. pteronyssinus*, only for monosensitized patients we used *D. farinae*. Moreover, patients reported their symptoms regarding secretion, irritation, and remote symptoms after each measurement and a symptom score was calculated (secretion: no secretion: 0 points, a little secretion: 1 point, a lot of secretion: 2 points, irritation: 0–2 times sneezing: 0 points, 3–5 times sneezing: 1 point, > 5 times sneezing: 2 points, and remote symptoms: no remote symptoms: 0 points, lacrimation and/or itching of the palate and/or itching of the ears: 1 point, conjunctivitis and/or chemosis and/or urticaria and/or cough and/or dyspnea: 2 points) [27]. NPT was considered positive for patients who showed either a decrease in airflow of > 40% at 150 Pa on the allergen-challenged side or a symptom score of > 3, or a combination of a symptom score of > 2 and a reduction in airflow of > 20%. Secretion, irritation, and remote symptoms were semiquantitatively assessed to determine the symptom score. In patients with negative NPT results despite strong evidence in the history in favor of a clinically relevant HDM allergy, NPT was repeated with a different provocation test solution (Allergopharma GmbH, Reinbek, Germany). We controlled patients 30 min after NPT for security reasons. We did not observe any severe allergic reactions to NPT. As allergic late phase reactions of the nose have been described, we recommended our patients a self-observation of their symptoms during 24 h.

### Statistical analysis

The statistical analysis was performed with SPSS 23.0 (SPSS Inc., Chicago, Illinois). For descriptive statistics, median values and the standard deviation were used. For comparisons between different groups, Fisher’s exact test was applied. Differences were considered statistically significant at *p* < 0.05.

## Results

Based on the above-mentioned inclusion criteria, the database search yielded 471 patients with proven sensitization to *D. pteronyssinus* or *D. farinae*. 90% of patients were sensitized to *D. pteronyssinus* and 93% were sensitized to *D. farinae*. 85% were sensitized to both, *D. pertonyssinus* and *D. farinae*. Only 6% of the individuals were monosenitized to *D. farinae*. The study population was divided into two groups: 248 patients with a positive NPT result to *D. pteronyssinus* or *farinae* and 223 with a negative NPT result.

Important for statistical analysis, the distribution of mono- (sensitization to one allergen only) and polysensitization (sensitization to more than one allergen) was comparable in both groups. Detailed demographic data of our study population are summarized in Table 1.

**Table 1:** Demographic data of patients allergic to HDM and patients with clinically silent sensitization

Characteristics	Negative NPT (*n* = 223)	Positive NPT (*n* = 248)	Total (*n* = 471)	Significance
Gender				0.701
Male	131 (59%)	150 (61%)	281 (60%)	
Female	92 (41%)	98 (40%)	190 (40%)	
Age	34 (16; 6–74)	29 (14; 5–81)	32 (17; 5–81)	0.610
Sensitization				0.717
Monosensitized to HDM	57 (27%)	61 (26%)	118 (26%)	
Polysensitized	153 (73%)	177 (74%)	330 (74%)	
Time of presentation				0.326
Winter (October–March)	105 (47%)	128 (52%)	233 (50%)	
Summer (April–September)	118 (53%)	120 (48%)	238 (50%)	

Age is given as median (standard deviation, minimum and maximum)

We found a trend that patients allergic to HDM complain more often about perennial symptoms than subjects with clinically silent sensitization (34% vs. 30%, *p* = 0.374).

Concerning nasal symptoms, as reported by patients in the questionnaire recording medical history, rates of nasal airway obstruction were comparable in both groups (66% vs. 69%, *p* = 0.630). Patients allergic to HDM tend to suffer more often from nasal secretion (45% vs. 37%, *p* = 0.244) but significantly less from nasal dryness (34% vs. 46%, *p* = 0.046).

With regard to ocular complaints, significantly more patients only sensitized to HDM reported about red eyes than patients with positive NPT to HDM (13% vs. 4%, *p* = 0.008). Moreover, within the group of polysensitized participants, significantly more patients with negative NPT to HDM suffered from red eyes (14% vs. 4%, *p* = 0.017). The rate of ocular itching was comparable in both groups (39% vs. 40%, *p* = 0.834). However we observed the trend that patients in the monosensitized subgroup with positive NPT reported more often about this condition (28% vs. 20%, *p* = 0.357).

Concerning atopic co-morbidities, the prevalence of self-reported asthma was comparable in both groups (20% vs. 19%, *p* = 0.851). However, in the subgroup of monosensitized patients, individuals allergic to HDM complained more frequently about asthma (0% vs. 5%, *p* = 0.061), whereas there was no single asthmatic person among patients with a clinically silent monosensitization to HDM. Patients in the positive NPT group suffered significantly more often from atopic dermatitis (14% vs. 7%, *p* = 0.046).

With regard to sleep quality, patients with clinically silent sensitization to HDM tended to be more often affected by un-restorative sleep (44% vs. 38%, *p* = 0.293). Symptoms in the morning were comparable between both patient groups: only slightly more patients allergic to HSM complained about symptoms in the morning (51% vs. 49%, *p* = 0.724). Markedly, but not significantly more patients with silent sensitization reported about snoring (33% vs. 23%, *p* = 0.072). However, the number of patients reporting about a dry mouth at night or in the morning was equal (44% vs. 40%, *p* = 0.563). All clinical data of the study population obtained are summarized in Table 2.

**Table 2:** Clinical data of patients allergic to HDM and patients with clinically silent sensitization (data taken from questionnaire recording medical history)

Characteristics	Negative NPT (*n* = 223)	Positive NPT (*n* = 248)	Total (*n* = 471)	Significance
Perennial complaints	66 (30%)	83 (34%)	146 (32%)	0.374
Location-dependent symptoms	60 (31%)	76 (39%)	136 (35%)	0.090
Nasal complaints				
Nasal airway obstruction	102 (69%)	108 (66%)	210 (68%)	0.630
Nasal secretion	53 (37%)	73 (45%)	126 (41%)	0.244
Nasal dryness	67 (46%)	54 (34%)	121 (40%)	0.046
*Ocular complaints*				
Red eye	17 (13%)	6 (4%)	23 (8%)	0.008
Monosensitized to HDM	2 (8%)	1 (3%)	3 (5%)	0.556
Polysensitized	15 (14%)	5 (4%)	20 (9%)	0.017
Itching	73 (40%)	78 (39%)	151 (39%)	0.834
Monosensitized to HDM	10 (20%)	14 (28%)	24 (23%)	0.357
Polysensitized	63 (48%)	64 (42%)	127 (45%)	0.339
Atopic dermatitis	8 (7%)	20 (14%)	28 (11%)	0.046
Asthma	14 (19%)	22 (20%)	36 (19%)	0.851
Monosensitized to HDM	0 (0%)	5 (17%)	5 (9%)	0.061
Polysensitized	14 (26%)	17 (21%)	31 (23%)	0.541
Sleep quality				
Un-restorative sleep	62 (44%)	61 (38%)	123 (41%)	0.293
Dry mouth at night	58 (40%)	70 (44%)	128 (42%)	0.563
Snoring	46 (33%)	38 (23%)	84 (28%)	0.072

In Table 3, additional information about the patients’ home environment is listed. Patients allergic to HDM seem to have suffered more often from location-dependent symptoms than subjects with clinically silent sensitization (39% vs. 31%, *p* = 0.090). While the age of the mattresses used by the patients of both main groups was comparable (39% vs. 37%, *p* = 0.723), patients allergic to HDM used encasings for their mattresses and pillows (23% vs. 18%, *p* = 0.127) more frequently. In Table 4, laboratory characteristics are listed.

**Table 3:** Additional information about patients’ home environment

Characteristics	Negative NPT (*n* = 223)	Positive NPT (*n* = 248)	Total (*n* = 471)	Significance
Location-dependent symptoms	60 (31%)	76 (39%)	136 (35%)	0.090
New mattress (≤ 2 years)	54 (37%)	61 (39%)	115 (38%)	0.723
Encasings	36 (18%)	53 (23%)	89 (21%)	0.127

**Table 4:** Laboratory characteristics

Laboratory characteristics	Positive NPT (*n* = 223)		Negative NPT (*n* = 248)	Total (*n* = 471)
Total IgE (kU/L)	320.98 (573.89)	284.66 (423.42)		303.72 (507.78)
*D. pter*				
CAP class	2.80 (1.40)	1.84 (1.34)		2.33 (1.45)
Serum IgE (kU/L)	16.56 (24.94)	6.91 (17.11)		12.06 (22.15)
*D. far*				
CAP class	2.70 (1.57)	1.63 (1.45)		2.17 (1.60)
Serum IgE (kU/L)	18.89 (29.21)	6.60 (16.20)		13.13 (24.79)

Data are given as mean (standard deviation)

## Discussion

Taking a detailed patients’ history is a crucial part of the process of finding the correct diagnosis in allergic diseases [10]. In case of allergy to HDM, experts in the field of allergy and immunology recommend additional NPT besides the common practice of taking medical history and evaluating the SPT and/or specific IgE level before starting allergy immunotherapy [13, 28]. A significant reason for this recommendation is that contrary to other aeroallergens such as pollen or pets, the symptoms patients allergic to HDM report of are considered as inconsistent. However, NPT has several disadvantages like time costs and induction of potentially harmful allergic reactions. In contrast to many evidence-based approaches in the field of allergy and immunology, to our knowledge, it has not been evaluated so far if it is possible to diagnose allergy against HDM based on the history only. Thus, the aim of this study was to evaluate retrospectively if the application of a standardized questionnaire alone—addressing the most relevant symptoms of AR—would be able to identify individuals with clinical relevant allergy to HDM.

Sensitization can be differentiated into monosensitization (sensitization against a single allergen) and polysensitization (sensitization against at least two allergens) [8]. In our study, sensitization was indicated either by a positive reaction on standardized SPT or positive serum sIgE-levels or both. According to epidemiological studies, polysensitization is more common than monosensitization [29, 30]. This was also the case in our preselected cohort, since only 26% of all participants were monosensitized to HSM.

Encasings were prescribed for patients with positive a NPT result. Other typical measures against HSM (including removal of carpets and indoor plants) were recommended. In case they had no contraindications, patients were treated with a nasal cortisol spray. All patients were asked to make another appointment in our department after three months to reevaluate their complaints. In case no sufficient improvement with above-mentioned therapy measures was seen and patients had no contraindications, allergen immunotherapy was recommended.

Perennial rhinitis, which is a typical characteristic of HDM allergy, is most often defined by the persistence of at least two of the following symptoms over nine months: serous or seromucous hypersecretion, nasal blockage caused by a swollen nasal mucosa, or sneezing paroxysms. Nasal congestion and mucous production (postnasal drip) are also predominant in most patients, while sneezing, itching, and watery rhinorrhea may be minimal [31, 32].

Although nasal obstruction is one of the main symptoms affecting the quality of life of adolescents [33] and adults [34] suffering from perennial AR, we found no relevant difference in the degree of nasal obstruction reported by the participants of both groups. Nasal secretion, however, was a little bit more pronounced in our patients with positive NPT (45% vs. 37%). Accordingly, nasal dryness was reduced within this group (34% vs. 46%). Nasal operations might influence nasal symptoms, especially nasal obstruction. In the presented study, we excluded patients with a history of nasal surgery less than 2 months prior to their presentation at our department. However, even earlier operations might have an impact on nasal symptoms. This might be a disadvantage of the study. Nevertheless, we assume that the influence of these operations on our data may be neglected, as we as we included a relatively large number of patients in both subgroups.

Our participants were also asked about their ocular complaints, comprising symptoms of bilateral red eyes but also itching and tearing of their eyes in general. Didier et al. reported in their cross-sectional observational survey about AR-associated ocular symptoms in 19% of the overall study population and 52% in the AR population of adults. Interestingly, HDM sensitization was one of the most important trigger factors of ocular symptoms (35%) identified in this study [35]. Another cross-sectional study demonstrated that a physician diagnosis of conjunctivitis was in 16% of 1549 asthmatic children (mean age 4.3 years). 44% of these children had at least one ocular symptom (itching, lacrimation or redness) suggesting ocular allergy. Again, HDM were with 71.4% one of the most common sensitizing agents [36]. Interestingly, in our study significantly more patients with silent sensitization against HDM reported about symptoms of conjunctivitis than individuals with clinically relevant allergy (13% vs. 4%). This effect was especially pronounced in the polysensitized group (14% vs. 4%). A possible explanation for this finding might be, that polysensitized patients suffered from red eyes because of other allergens. However, the rate of ocular itching was comparable in both groups (39% vs. 40%).

In summary, our questionnaire revealed no suitable differences in nasal or ocular symptoms between subjects with relevant allergy against HDM and individuals with silent sensitization, even though these findings are known to belong to the key symptoms of AR. Concerning the temporal and regional occurrence of allergic symptoms, we did not observe significant differences (see Table 2).

We also detected no difference in the prevalence of self-reported asthma between the two groups (20% vs. 19%). Overall, this is a much higher prevalence compared to the prevalence of asthma in Germany, reported to be 6.2% [37], which is in line with the fact that asthma is more frequently seen in patients with allergen sensitization. In the monosensitized subgroup, there was no patient with asthma in the silent sensitization group, but five patients with asthma and allergy to HSM (0% vs. 17%). This finding shows that there is a connection between allergy to HDM and asthma.

Besides causing allergic rhinitis and asthma, dust mite allergens are known to induce further atopic diseases such as atopic dermatitis (AD) [38]. AD is characterized by pruritus and chronic or relapsing eczematous lesions with typical morphology and distribution [39]. Several studies demonstrated a relationship between the exposure to high levels of HDM allergens in babyhood and the development of asthma [7, 40] and atopy [41] in childhood. Accordingly, Zureik et al. found in a huge cross-sectional study a positive association between sensitization to HDM and the severity of asthma [42]. Besides inhalation, a possible route of exposure to HDM allergens is direct contact with the skin [43, 44]. Consequently, it is not surprising that significantly more patients of our cohort, allergic to HDM, suffered from these atopic diseases in contrast to the control group of sensitized only patients (14% vs. 7%), underlining some representative character of our study population. The differences between the two groups were statistically significant but these items of our questionnaire alone are not useful for the differentiation between clinical relevant HDM allergy and pure sensitization.

A further part of the questionnaire deals with the possible impact of AR on different facets of sleep, comprising interruption of the sleep, un-restorative sleep, snoring, and dry mouth at night or in the morning. It is known that AR negatively affects sleep in many ways. This was confirmed by a meta-analysis of observational studies [45]. Nasal congestion, which is a typical symptom of AR, is a known risk factor for sleep-disordered breathing and snoring [46–48]. Thus, application of nasal corticosteroids positively affects sleep quality in patients with AR [49, 50]. In contrast, in the presented study, there were no significant differences in the severity of symptoms related to sleep between the patients with HDM allergy and those with sensitization. Regarding some aspects (un-restorative sleep and snoring) individuals with silent sensitization tended to suffer slightly more often from reduced sleep quality. Other diseases also might influence sleep quality. These were not excluded in this study, which might influence the presented data. However, as we included a relatively large number of patients in both subgroups, we assume that this effect can be neglected.

The last part of our questionnaires addresses the home environment of the participants. This is especially important in case of HDM allergy because the bed and the mattresses are essential habitats for mites [2, 51]. Factors shown to decrease HDM concentrations in the home include use of newer mattresses and mite-impermeable mattress covers [52, 53]. Interestingly, more patients allergic to HDM reported about location-dependent complaints (39% vs. 31%) and more often used encasings for their beds (23% vs. 18%). In contrast, the age of the mattresses possessed by both groups was comparable. One could speculate that the individuals affected by HDM allergy have already investigated their symptoms and tried to start action against them thereby emphasizing the pre-selection of the cohort presenting at a center of maximum care as our university hospital.

There are several discrepancies between the findings presented in this study and the cited results found in the literature. Some of this could be explained by different inclusion criteria and initial questions. Moreover, our study has a pre-selection bias, as all patients presented at a specialized university clinic due to upper airway complaints. Thereby this cohort is not representing the general population. However, patients presenting to a specialist in allergy and clinical immunology would have similar characteristics, underlining the importance of the questions we address. Besides this main limitation, slight additional drawbacks of our study have to be addressed. First, our questionnaire was adapted from the RQLQ [20, 21]. It is a standardized but not a validated tool. Furthermore, some items are related to the last 7 days, which may lead to false negative results, for example if patients do not present in the main allergy season. Moreover, the questionnaire addressed subjective symptoms. Objective data, for example pulmonary function tests, was not collected. Last, all data were evaluated retrospectively. In spite of these limitations, the presented study shows important data on the concordance between anamnestic data and NPT in HDM sensitized patients. It confirms the current opinion that clinical data in HDM sensitized patients is insufficient for initiating allergen-specific immunotherapy.

## Conclusion

Taken together, the results of our study corroborate the common concept in the field of allergy and immunology that taking patient’s history alone seems not to be a suitable instrument for finding the correct diagnosis of allergy to HDM. As a consequence, further steps like nasal allergen challenge recommended by experts and known from the guidelines are still reasonable and inevitable.
